# Clinical Data on Daptomycin plus Ceftaroline versus Standard of Care Monotherapy in the Treatment of Methicillin-Resistant Staphylococcus aureus Bacteremia

**DOI:** 10.1128/AAC.02483-18

**Published:** 2019-04-25

**Authors:** Matthew Geriak, Fadi Haddad, Khulood Rizvi, Warren Rose, Ravina Kullar, Kerry LaPlante, Marie Yu, Logan Vasina, Krista Ouellette, Marcus Zervos, Victor Nizet, George Sakoulas

**Affiliations:** a Sharp Memorial Hospital, San Diego, California, USA; b Sharp Grossmont Hospital, La Mesa, California, USA; c Henry Ford Hospital, Detroit, Michigan, USA; d University of Wisconsin School of Pharmacy, Madison, Wisconsin, USA; e Doctor Evidence, LLC, Santa Monica, California, USA; f University of Rhode Island College of Pharmacy, Kingston, Rhode Island, USA; g University of California San Diego School of Medicine, La Jolla, California, USA

**Keywords:** bacteremia, ceftaroline, daptomycin, methicillin-resistant *Staphylococcus aureus*, mortality, vancomycin

## Abstract

Vancomycin (VAN) and daptomycin (DAP) are approved as a monotherapy for methicillin-resistant Staphylococcus aureus (MRSA) bacteremia. A regimen of daptomycin plus ceftaroline (DAP+CPT) has shown promise in published case series of MRSA salvage therapy, but no comparative data exist to compare up-front DAP+CPT head-to-head therapy versus standard monotherapy as an initial treatment. In a pilot study, we evaluated 40 adult patients who were randomized to receive 6 to 8 mg/kg of body weight per day of DAP and 600 mg intravenous (i.v.) CPT every 8 h (q8h) (*n* = 17) or standard monotherapy (*n* = 23) with vancomycin (VAN; dosed to achieve serum trough concentrations of 15 to 20 mg/liter; *n* = 21) or 6 to 8 mg/kg/day DAP (*n* = 2). Serum drawn on the first day of bacteremia was sent to a reference laboratory *post hoc* for measurement of interleukin-10 (IL-10) concentrations and correlation to in-hospital mortality. Sources of bacteremia, median Pitt bacteremia scores, Charlson comorbidity indices, and median IL-10 serum concentrations were similar in both groups. Although the study was initially designed to examine bacteremia duration, we observed an unanticipated in-hospital mortality difference of 0% (0/17) for combination therapy and 26% (6/23) for monotherapy (*P* = 0.029), causing us to halt the study. Among patients with an IL-10 concentration of >5 pg/ml, 0% (0/14) died in the DAP+CPT group versus 26% (5/19) in the monotherapy group (*P* = 0.057). Here, we share the full results of this preliminary (but aborted) assessment of early DAP+CPT therapy versus standard monotherapy in MRSA bacteremia, hoping to encourage a more definitive clinical trial of its potential benefits against this leading cause of infection-associated mortality. (The clinical study discussed in this paper has been registered at ClinicalTrials.gov under identifier NCT02660346.)

## Abstract (precis)

Vancomycin (VAN) and daptomycin (DAP) are approved as a monotherapy for methicillin-resistant Staphylococcus aureus (MRSA) bacteremia. A regimen of daptomycin plus ceftaroline (DAP+CPT) has shown promise in published case series of MRSA salvage therapy, but no comparative data exist to compare up-front DAP+CPT head-to-head therapy versus standard monotherapy as an initial treatment.

## INTRODUCTION

Methicillin-resistant Staphylococcus aureus (MRSA) bacteremia is associated with a significant disease burden and a high case fataility, ranging from 20% to 30%, which is double that seen in methicillin-susceptible S. aureus (MSSA) bacteremia (1–3). This and other metrics of poor outcomes in patients with MRSA bacteremia are attributed to inferior pharmacotherapeutic properties of vancomycin (VAN), the cornerstone of MRSA therapy, compared with β-lactam antibiotics used to treat MSSA bacteremia (4, 5). Clinical studies demonstrating such differences have been bolstered by experimental data showing that β-lactams not only exert direct antibacterial effects on S. aureus but also synergize with cationic antimicrobial peptides and other arms of the innate immune system to promote pathogen clearance (6). These secondary effects may be noteworthy enough that the addition of antistaphylococcal β-lactams (oxacillin, nafcillin, and flucloxacillin) to cornerstone daptomycin (DAP) and VAN can aid in the clearance of refractory MRSA bacteremia, despite the β-lactam agents having no direct anti-MRSA activity, as measured by standard susceptibility testing methods (7, 8).

Based on a study showing noninferiority of DAP to VAN in treating MRSA bacteremia (9), the Infectious Diseases Society of America (IDSA) 2011 MRSA treatment guidelines recommend initiating one of these two agents as first-line MRSA bacteremia therapy (10). Although definitions vary, initial treatment failure is encountered in up to 50% of cases and is linked to poor outcomes, including a greater likelihood of metastatic infections and increased mortality (11–13). The IDSA guidelines recommend switching to an alternative regimen for persistent MRSA bacteremia of ≥7 days or earlier if clinical deterioration is evident (10). Case reports and series have documented high success in the treatment of persistent MRSA bacteremia by combining DAP with antistaphylococcal β-lactams or ceftaroline (CPT) (14). Potential mechanisms underlying this advantageous dual therapy are (i) β-lactam reduction of cell wall cross-linking, enhancing DAP access to the cell membrane (14); (ii) synergy of β-lactams with endogenous cationic host defense peptides against MRSA (6); and (iii) increased NLRP3 inflammasome activation and interleukin-1β (IL-1β)-mediated bacterial clearance induced by altered peptidoglycan synthesized by MRSA under β-lactam challenge (15, 16). Despite excellent clinical responses to DAP+CPT as salvage therapy in difficult MRSA bacteremia cases, immediate initiation of DAP+CPT has not been compared with the current standard of care monotherapy. Furthermore, data are emerging that suggest that VAN monotherapy may be insufficient to treat severe MRSA respiratory infections, whereby a second agent may be needed to the improve outcome (17).

We prospectively examined DAP+CPT within 72 h of bacteremia onset to standard of care VAN or DAP monotherapy in the treatment of MRSA bacteremia in 3 hospital centers. Due to the fact that the study was not carried out in a blind manner and because an unexpected mortality difference was identified before completion, the study was halted early and the cohort of patients remained small. Serum concentrations of interleukin-10, a strong predictor of mortality in S. aureus bacteremia (15, 18, 19), were evaluated in a blind manner by a reference laboratory *post hoc* to identify a high-risk patient subset for whom the clinical benefit of early DAP+CPT therapy may be most pronounced. We present these potentially “hypothesis generating” data to encourage a larger prospective, randomized, blind clinical trial of combination antimicrobial therapy in the treatment of MRSA bacteremia.

## RESULTS

### Baseline characteristics.

Forty patients over 18 months at 3 hospitals (see Fig. S1 in the supplemental material) were prospectively assigned at random to receive DAP+CPT (*n* = 17) or monotherapy with VAN (*n* = 21) or DAP (*n* = 2). Their baseline clinical characteristics and comorbidities are shown in Table 1. The groups did not differ significantly in terms of distribution of age, sex, comorbidities, overall comorbid status, or severity of illness, although trends for some specific comorbidities were weighted in one group versus the other. For example, 18% of the combination group had a history of stroke versus 0% in the monotherapy group (*P* = 0.07). Liver disease was also more prevalent in the combination group (29% versus 9%, *P* = 0.11). Chronic lung disease trended more in the monotherapy group over the combination group (52% versus 24%, *P* = 0.10). Cancer was also weighted more in the monotherapy group (22% versus 0%, *P* = 0.06). The median Pitt bacteremia score was 1 for both groups. The age-adjusted median Charlson comorbidity index was 5.0 in the combination group and 6.0 in the monotherapy group (*P* = 0.52); 12% (2/17) of the combination therapy patients and 13% (3/23) of the monotherapy patients were admitted to the intensive care unit at the time of infection.

**TABLE 1:** Patient demographics and characteristics^*a*^

Characteristic^*b*^	Values by treatment:	
	Daptomycin plus ceftaroline (*n* = 17)	Vancomycin (*n* = 21) or daptomycin (*n* = 2)
Male, *n* (%)	9 (53)	16 (70)
Mean age (yr)	62	62
Mean BMI	30.7	26.7
Comorbidities, *n* (%)		
Cardiovascular Dz	9 (53)	10 (43)
Diabetes mellitus	6 (35)	11 (48)
Cerebrovascular Dz	3 (18)	0 (0)
End-stage renal Dz	3 (18)	6 (26)
Immunocompromised	0 (0)	1 (4)
Chronic lung Dz	4 (24)	12 (52)
Severe liver Dz	5 (29)	2 (9)
Malignancy	0 (0)	5 (22)
Neutropenia	0 (0)	1 (4)
Charlson index		
Mean	4.7	5.5
Median	5	6
Pitt bacteremia score		
Mean	1.47	1.09
Median	1	1
Acute renal failure, *n* (%)	3 (18)	5 (22)
Intensive care, *n* (%)	3 (18)	3 (13)

a *P* values are >0.05 for all comparisons.

b BMI, body mass index; Dz, disease.

### Bacteremia sources.

The primary sources of bacteremia (endovascular noncatheter versus extravascular versus catheter) were evenly distributed among the treatment groups (Table 2). Foci of infection (more than one may be present per patient) are also shown in Table 2. Overall, infections were evenly distributed among the groups, except for a trend toward more vascular catheter infections in the monotherapy group compared with the combination therapy group (13% versus 0%, *P* = 0.12). Most infections (37/40) were identified ≤72 h within admission.

**TABLE 2:** Sites of infection^*a*^

Source^*b*^	Values by treatment, *n* (%):	
	Daptomycin plus ceftaroline (*n* = 17)	Vancomycin (*n* = 21) or daptomycin (*n* = 2)
1° Bacteremia source		
Endovascular	8 (47)	8 (35)
Secondary tissue	9 (53)	12 (52)
Catheter	0 (0)	3 (13)
Foci of infection present		
Venous catheter	1 (6)	3 (13)
Urinary tract	3 (18)	4 (17)
Respiratory tract	1 (6)	6 (26)
Surgical wound	0 (0)	2 (9)
Skin/soft tissue	9 (53)	8 (35)
Bone/joint	5 (29)	4 (17)
LVAD	1 (6)	1 (4)
Intra-abdominal	0 (0)	2 (9)
Endocarditis plus TEE	3 (18)	1 (4)

a All values are *n* (%). *P* values are >0.05 for all comparisons.

b LVAD, destination left ventricular assist device; TEE, transesophageal echocardiogram.

### Laboratory data.

Relevant laboratory data in the two patient groups are shown in Table 3. There were no differences in leukocyte count, platelet counts, calculated creatinine clearance, and C-reactive protein between the groups. Median admission interleukin-10 (IL-10) concentrations were 9.5 pg/ml and 7 pg/ml for the combination therapy and monotherapy groups, respectively. All MRSA isolates where combination therapy was used had CPT and DAP MICs of ≤0.5 mg/liter and a VAN MIC of ≤1 mg/liter according to the clinical microbiology laboratory.

**TABLE 3:** Relevant laboratory and treatment data^*a*^

Metric^*b*^	Values by treatment type:	
	Combination therapy	Monotherapy
Vancomycin MIC (mg/liter), *n* (%)		
0.5	5 (29)	3 (9)
1	12 (71)	20 (91)
2	0 (0)	0 (0)
Blood analyses, median (IQR)		
WBC (×1,000/mm^3^)	17.3 (13.7, 22.6)	14.2 (9.7, 18.5)
Platelet (×1,000/mm^3^)	246 (144, 384)	173 (98, 323)
CrCl (ml/min)	74 (24, 119)	47 (16, 114)
Procalcitonin (ng/ml)	0.22 (0.07, 0.74)	0.72 (0.52, 8.8)
CRP (mg/liter)	127 (109, 212)	176 (108, 236)
IL-10 (pg/ml)	9.5 (5, 20.5)	7 (5.5, 20.5)
Treatment		
Vancomycin trough (initial, mg/liter)	N/A^*c*^	16.2 (10.7, 19.8)
Daptomycin dose (median, mg/kg)	8.6	8.0

a *P* values are >0.05 for all comparisons.

b IQR, interquartile range; WBC, white blood cell; CRP, C-reactive protein.

c Values were obtained for only 3 patients prior to randomization with initial trough values (mg/liter), namely, 27, 19, and 9. N/A, not available.

### Treatments.

The 23 standard of care patients received a median duration of randomization therapy of 12 days and a total duration of therapy of 26 days. The combination therapy arm received DAP+CPT for a median of 8 days and a mean of 11 days and a total median treatment duration of 38 days (Fig. S1). The median time to randomization from onset of bacteremia to initiation of study therapy was 2 days.

### Outcomes.

Relevant outcomes are listed in Table 4. The median bacteremia duration was 3 days for each group. In-hospital mortality was 0% (0/17) for combination therapy and 26% (6/23) for monotherapy (*P* = 0.029). Among patients with an admission IL-10 concentration of ≤5 pg/ml, in-hospital mortality was 0% (0/3) for the combination group and 25% (1/4) in the monotherapy group (*P* = 1.0). For an IL-10 concentration of >5 pg/ml, in-hospital mortality was 0% (0/14) in the combination therapy group versus 26% (5/19) in the monotherapy group (*P* = 0.057).

**TABLE 4:** Study outcomes

Outcome	Values by treatment type:		*P* value
	Combination therapy	Monotherapy	
Mortality, *n* (%)			
In hospital	0 (0)	6 (26)	0.02
30 day	0 (0)	6 (26)	0.02
90 day	0 (0)	7 (30)	0.03
Bacteremia duration, median (IQR) days	3 (1.5, 5.5)	3 (1, 5.3)	0.56
Length of stay, median (IQR) days	11 (6, 14)	12 (8, 23)	0.24

**FIG 1:**
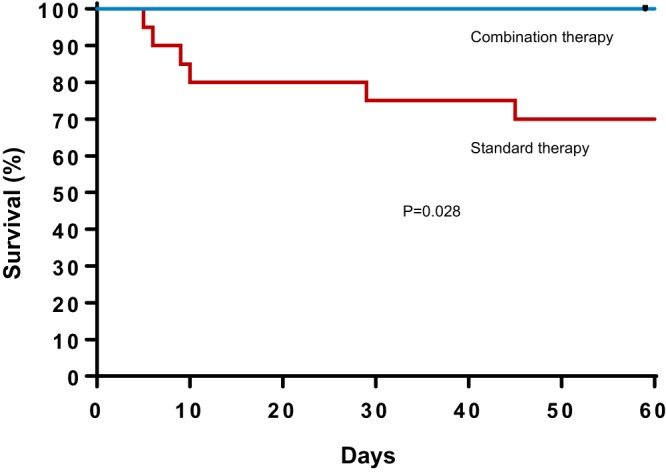
Survival analysis of patients receiving daptomycin plus ceftaroline compared with those receiving standard of care in a prospective randomized study. Day 0 represents the day of first positive blood culture. Significance of mortality difference at 30 days (*P* = 0.048) and 60 days (*P* = 0.028).

Notably, but perhaps not surprisingly, mortality lay entirely within the patient cohort with endovascular sources of primary infection. A subanalysis of this subgroup (called for by expert reviewers) is shown in Table S1 in the supplemental material and detailed clinical descriptions in Table S2 in the supplemental material. Most of these cases were of left-sided endocarditis cases, including those with prosthetic valves and intracardiac devices, such as left-ventricular assist devices (LVADs) and intracardiac defibrillators (ICDs). A Kaplan-Meir 60-day survival analysis of the two treatment groups overall is shown in Fig. 1, demonstrating a significantly increased risk of mortality in the standard of care group compared with the combination therapy group.

### Treatment-related adverse events.

A summary of adverse events is provided in Table 5. Three patients that received monotherapy treatment were salvaged with combination therapy due to treatment failure after 5 days. Of these three, one patient survived, a second died in the hospital, and the third died from pneumonia due to extended-spectrum β-lactamase (ESBL)-producing Escherichia coli during a follow-up admission occurring 2 months after the initial presentation. One patient with VAN therapy developed acute renal failure attributable to VAN. One DAP monotherapy patient developed asymptomatic elevation in creatinine phosphokinase (CPK) 13 days into therapy, prompting DAP discontinuation and completion of therapy with 2 additional weeks of CPT. One combination patient was de-escalated to DAP monotherapy upon discharge from the hospital but developed eosinophilic pneumonia. Therapy was changed to CPT monotherapy, which was administered for an additional 4 weeks. Another combination therapy patient was de-escalated at 4 weeks to CPT monotherapy, with a plan to complete 2 more weeks. However, the patient developed worsening mitral regurgitation, increasing C-reactive protein (CRP) and concern for pneumonia 5 days after this de-escalation, prompting a switch to telavancin. After a week of telavancin, an increase in creatinine prompted another switch to linezolid, which was administered for 1 more week until CRP normalization.

**TABLE 5:** Treatment-related adverse events

Event	No. of patients	
	Combination therapy	Monotherapy
Treatment failure	1^*a*^	3^*b*^
Acute kidney injury	0	1
Asymptomatic elevated CPK^*c*^	0	1
Eosinophilic pneumonia	1^*d*^	0

a Occurred after de-escalation to ceftaroline monotherapy.

b Early failure prompting switch to combination therapy at day 5 of therapy.

c CPK, creatine phosphokinase.

d Occurred after de-escalation to daptomycin monotherapy.

## DISCUSSION

Since its emergence as a common nosocomial pathogen in the 1990s, MRSA mortality and duration of bacteremia were noted to be almost twice that experienced with MSSA infections (4). While the reasons for this disparity were not initially clear, recent insights about the pharmacodynamic synergy of β-lactams with the innate immune system suggested that the absence of such effects among non-β-lactam drug repertoire available for MRSA treatment may be responsible (6). The consistent inferior performance of VAN compared with β-lactams in MSSA bacteremia (5, 20–23), coupled with the negative clinical prognosis conferred upon patients with β-lactam drug allergies who are denied β-lactam treatment (24–27), appeared to reinforce this conclusion.

Clinical success has been reported when using DAP+CPT as a salvage therapy in treating refractory MRSA bacteremia (14). This pilot study was performed to compare the current standard of care for MRSA bacteremia (VAN or DAP monotherapy) with DAP+CPT. Higher mortality was seen in patients with endovascular infections and in patients with serum IL-10 concentrations of >5 pg/ml who were treated with monotherapy compared with DAP+CPT.

The median duration of bacteremia was similar in both groups. A review of these cases, as well as the data from the CAMERA-1 study (8), demonstrated the presence of outlier cases of increased duration that may not be captured in median calculations. In the CAMERA-1 study, outliers with prolonged bacteremia of >4 days were noted in 13 of 49 (27%) VAN monotherapy patients versus only 3 of 46 (7%) in the VAN plus flucloxacillin group, a difference that is statistically significant (*P* = 0.01, Fisher’s exact). Therefore, eliminating or reducing the high-risk outlier patients with prolonged MRSA bacteremia represents a great medical need of antimicrobial pharmacotherapy that may be met with the addition of β-lactam combination therapy. In this small study, 3 of 23 patients in the monotherapy arm (13%) were salvaged by combination therapy due to bacteremia duration of ≥5 days.

While the DAP+CPT combination therapy was very well tolerated overall in this small cohort, one of its most significant limitations is drug cost. The drug acquisition costs of DAP+CPT at the doses employed in this study are about $760/day, a cost at least 10-times that of VAN (28). However, it is important to highlight that only a fraction of the treatment duration in the combination therapy group (median, 8 days; mean, 11 days) was with the combination regimen. De-escalation was almost universally adopted (including 3 patients de-escalated to oral oxazolidinone therapy), in large part because disposition would have been very difficult on this cumbersome and expensive regimen. Therefore, an up-front cost of about 10 days of combination therapy, followed by de-escalation (e.g., VAN) may be more economical than an up-front treatment failure on monotherapy that requires salvage by a more expensive antimicrobial combination regimen. Nevertheless, the most cost-effective antimicrobial regimen may well lie in a more “intermediate” ground, such as VAN plus CPT or DAP plus an antistaphylococcal β-lactam or cefazolin. Note that while DAP has MRSA activity *in vitro*, our findings of the innate immune-boosting effects of nafcillin *in vivo* (6, 7) plus the fact that nafcillin can be given up to 12 g/day as opposed to only 1.8 g CPT raises the possibility that potentiation of DAP activity may be achieved in combination with a number of antistaphylococcal β-lactams and not exclusively with CPT. A larger study (CAMERA-2) is currently examining the use of flucloxacillin to VAN or DAP (29).

The cost effectiveness of early combination therapy in MRSA bacteremia may be further increased by risk stratification to preferentially allocate more cumbersome and expensive therapy to those in whom benefit would be greatest. This study employed a *post hoc* assessment of IL-10 serum concentrations from study participants on the day of initial blood culture and frozen upon patient enrollment. The samples were shipped to ARUP laboratories, and analyzed in a blind manner through a low-sensitivity assay that is readily available to all clinicians. This assay quantitates concentrations of IL-10 of ≥5 pg/ml, which approximates the previously published cutoff of 7.8 pg/ml determined by the ultrasensitive assay. As in previous studies, an elevated IL-10 was predictive of patient mortality in this study. A strong trend toward increased survival was seen in patients with MRSA bacteremia with IL-10 of > 5 pg/ml that received combination therapy compared with those that received monotherapy. There were not sufficient numbers of patients with IL-10 of <5 pg/ml in this study to draw meaningful conclusions in this subgroup.

This exploratory study has some very important limitations largely centered around its very small size and resulting random variability between the 2 groups. For example, randomization of 40 patients resulted in a 17 versus 23 in the treatment group sizes. A disproportionate number of cancer patients were randomized to the control monotherapy group, including 2 of them with stage IV lung cancer at the time of MRSA bacteremia onset, which may have adversely affected their outcomes. These patients with terminal cancer presented to the hospital and died with MRSA bacteremia as their main diagnosis, but readers may question whether the short-term in-hospital mortality could have been prevented with more aggressive antimicrobial therapy. These 2 patients had high-risk endovascular infections. Despite the small study size, all evaluated patients were enrolled, reflecting a “real-world” intent to treat a patient population, rather than a “cherry-picked” population wrought with exclusionary criteria. Lastly, we acknowledge that evaluating CPT monotherapy versus vancomycin in a similar prospective nature would be valuable to compare against our findings.

With the minimization of patient harm in mind, the moral dilemma posed by the disproportionate number of deaths in the standard group resulted in the early termination of this study, diminishing its statistical power and its scientific rigor, rendering it a pilot hypothesis generating study. Although this study was prospective and randomized, the open-label nature allowed the investigators full knowledge of the treatments administered and patient outcomes. This openly available information led to an early loss of equipoise due to serious concern for patient safety when a disproportionate number of deaths were occurring in the monotherapy arm.

In summary, this exploratory study showed with a very small number of patients that initial therapy with DAP +CPT may be associated with reduced in-hospital mortality compared with the treatment standards of VAN or DAP monotherapy in patients with MRSA bacteremia. The survival benefit, if any, may be limited to patients with high-risk endovascular sources and those with IL-10 of >5 pg/ml on the day of first positive blood culture. Given what is potentially at stake in this pre-eminent nosocomial infection with unacceptably high treatment failure rates, we strongly encourage a larger prospective study conducted in a blind manner to determine (i) the role of combination therapy, particularly with a β-lactam, in improving MRSA bacteremia outcomes; and (ii) employing biomarkers, such as IL-10, as potential risk stratification tools for allocating combination therapy to those at high risk.

## MATERIALS AND METHODS

### Study design.

Patients were randomized by computer assignment to their treatments in a prospective open-label manner. The original research protocol was registered on Clinicaltrials.gov on October 21, 2015 (registration no. NCT02660346) and subsequently reviewed, modified, and approved by the internal review boards of participating hospitals. Notable modifications were adjustment of mortality to the primary endpoint and the measurement of IL-10 at a reference lab rather than in our laboratory by a high-sensitivity research-grade enzyme-linked immunosorbent assay (ELISA). Patients were enrolled at Sharp Memorial Hospital (San Diego, CA) and Sharp Grossmont Hospital (La Mesa, CA) starting February 15, 2016, and at Henry Ford Hospital (Detroit, MI) on December 1, 2016. All participants provided written informed consent.

### Study population.

Adult patients (age, ≥18 years) with MRSA bacteremia were identified in the clinical microbiology laboratory by the Nanosphere Verigene Gram-positive blood culture assay (Luminex, Madison, WI). The infectious disease pharmacists and/or clinical investigational pharmacist were notified by the local laboratory to activate infectious disease consultation and enrollment procedures. All patients received an infectious disease consultation at the time of enrollment. Patients were excluded if they had arrived on transfer from another facility, had >72 h of pre-enrollment antibiotics, had polymicrobial bacteremia, or who were deemed to be terminally ill with comfort-only measures at the time of possible enrollment. All patients who were moribund (anticipated to die within 72 h of enrollment despite full therapy) were also excluded.

### Treatments.

Study participants were randomized at ≤72 h of initial blood culture. The study group received a combination of 6 to 8 mg/kg/day DAP plus 600 mg i.v. CPT q8h (adjusted per renal function). The control group received monotherapy with VAN (dosed by the clinical pharmacy service to achieve serum trough concentrations of 15 to 20 mg/liter) or 6 to 8 mg/kg/day DAP (adjusted for renal function). Blood cultures were obtained every 24 h until clearance, with a requirement of 2 consecutive days of negative cultures. Patients remained on the study regimen to which they were randomized for >4 days, but total duration of treatment was determined by the treating physician. At any point after 4 days (e.g., upon hospital discharge), the treating clinician had the option to select alternative antimicrobial therapy and duration appropriate for the disease state and disposition of the patient. If the patient was bacteremic for ≥5 days or deemed to be failing clinically on the regimen selected by the randomization process and a source control treatment option was not evident, the treating clinician had the option to resort to an alternative salvage regimen.

### Clinical data extraction and analysis.

At the time of enrollment, the following characteristics were recorded or calculated: patient age, weight, relevant comorbidities, location at bacteremia onset (community versus health care-associated), admission ward (intensive-care unit [ICU] or non-ICU), Charlson comorbidity index (https://www.mdcalc.com/charlson-comorbidity-index-cci; 30, 31), Pitt bacteremia score (32), and renal function (including chronic renal insufficiency, end-stage renal disease requiring dialysis, acute kidney injury; serum creatinine at time of first positive blood culture used to calculate CrCl via the Cockcroft-Gault method) (30). Subsequent data captured included CPT, DAP, and VAN MICs reported by the clinical microbiology laboratory (MicroScan automated broth microdilution; Beckman Coulter, Inc., Brea, CA), and the source of bacteremia was defined by the clinical work-up. Bacteremia was categorized into one of three risk categories based on prior literature: (i) primary endovascular bacteremia (e.g., proven or suspected endocarditis, presence of a retained intracardiac device such as left ventricular device or pacemaker, mycotic vessel, infected hemodialysis fistula or graft); (ii) secondary bacteremia from a primary tissue focus of infection (skin, bone/joint, pulmonary, or urine); or (iii) venous catheter-associated (33). A patient with bacteremia of >48-h duration on antibiotics without an identifiable focus was categorized as high-risk endovascular source/endocarditis, and treatment duration was established on this premise.

### Outcomes.

Primary outcomes examined were duration of bacteremia and in-hospital mortality. Secondary outcomes were later (60 and 90 day) mortality and length of hospital stay.

### Serum interleukin-10 measurement.

At the time of patient enrollment and randomization, serum collected on the day of the first positive blood culture as part of routine medical management was obtained from the clinical laboratory and frozen at −20°C in 0.5-ml aliquots. Samples were sent to ARUP reference laboratories (Salt Lake City, UT) for a *post hoc* measurement in a blind manner of IL-10 concentration via a quantitative multiplex bead assay (ltd.aruplab.com/Tests/Pub/0051534), and results were reported back to the principal investigator (PI; author G.S.) for analysis. This assay quantitates and reports IL-10 concentrations of ≥5 pg/ml, a value close to our previous cutoff of 7.8 pg/ml deemed to predict mortality (15). The ≥5-pg/ml cutoff was applied for mortality assessment in this study.

### Statistical analysis.

The planned enrollment sample size was 50 patients. However, after 40 patients were enrolled, the investigators perceived a mortality risk to the monotherapy patients, leading to an ethical obligation to halt the study on July 14, 2017. The data were then compiled and reviewed independently with two physician experts from other institutions who were not involved with study design or execution, including enrollment, and were not involved in the final outcome evaluation of the study. These physician experts provided the investigators with an unbiased perspective in the clinical management of these patients. These reviews occurred separately with each of the experts, with the question of whether the study ethically could continue by these investigators given the outcomes observed. Both experts independently agreed to halt the study due to the loss of equipoise with available data but that a larger and more comprehensive study conducted in a blind manner, including an independent data monitoring safety board, would be needed for a definitive answer in changing treatment standards. The study was officially terminated and the results reported herein.

All analyses were performed on the intent to treat the population. Statistical differences in mortality at the various time points and other categorical or ordinal variables were calculated using a 2-tailed Fisher’s exact test, and differences in continuous variables were calculated using the Mann-Whitney U test. Survival curves were generated with the Kaplan-Meier estimate method, and the log-rank test was used to compare standard therapy versus combination therapy survival at 60 days. For these comparisons, a *P* value of <0.05 was considered statistically significant.

## Supplementary Material

Supplemental file 1
