# Supplementary material for: Clinical Data on Daptomycin plus Ceftaroline versus Standard of Care Monotherapy in the Treatment of Methicillin-Resistant Staphylococcus aureus Bacteremia
Source: Antimicrob Agents Chemother. 2019 Apr 25;63(5):e02483-18. doi: 10.1128/AAC.02483-18 (PMC6496065; doi:10.1128/AAC.02483-18)
Supplement: Supplemental file 1 [file AAC.02483-18-s0001.pdf]

Supplemental Figure S1. Consort diagram describing the disposition of un-blinded randomization of the two treatment patient groups

40 patients with + MRSA Blood Culture
Identified By Verigene
Confirmed By Standard Microbiology Testing (MicroScan)

Randomization Within 72 hrs

17 Patients
Combination Therapy
Daptomycin + Ceftaroline

(All completed per protocol therapy)

Duration of Randomization Regimen (days)
Median (IQR): 8 (5,13)
Mean: 11

Duration of Total Treatment (days)
Median (IQR): 38 (16,46)
Mean: 34

23 Patients
Standard Monotherapy
Vancomycin n=21; Daptomycin n= 2

(3 patients switched to combination therapy for bacteremia $\geq$ 5 days)

Duration of Randomization Regimen (days)
Median (IQR): 12 (5,35)
Mean: 18

Duration of Total Treatment (days)
Median (IQR): 26 (17, 32)
Mean:27

Supplemental Table 1. Outcome of Primary Endovascular Patients

	Combination Therapy (N=8)	Monotherapy (N=8)	P Value
In Hospital Mortality	0 (0)	6 (75)	0.007
90-Day Mortality	0 (0)	7 (88)	0.001
Bacteremia Duration (days)			
Median (IQR)	5 (2, 7)	4 (4, 6)	
Mean	5.00	5.13	
Pitt Bacteremia Score			
Median (IQR)	2 (1, 8)	1 (0,3)	
Mean	3.71	1.6	
Charlson Score			
Median (IQR)	8 (3, 8)	7 (5.25, 10.5)	
Mean	7.125	7.38	

Supplemental Table 2. Clinical Details on 16 Endovascular Infection Patients

Age/Sex	Randomized Therapy	CrCl At Bacteremia Onset	In-Hospital Mortality	Pitt Score	Charlson Score	IL-10 (pg/mL)	Clinical Details
65/M	VAN	71	No	1	5	<5	Chronic TPN from prior perforated diverticulitis complications; resulting in CVL-associated right and left-sided endocarditis with septic pulmonary emboli, vertebral osteomyelitis of lumbar and cervical spine, sternoclavicular septic arthritis
53/M	VAN	22	No*	0	7	8	Infection of AV dialysis graft; prolonged MRSA bacteremia, failed to clear with 5 days VAN and AV graft debridement, cleared on DAP+CPT. Clinically deteriorated, transferred to ICU, developed *E. coli* HCAP; survived hospital discharge but readmitted and died of *E. coli* HCAP 2 months later
66/F	VAN	146	Yes	0	9	<5	Septic thrombophlebitis from a CVL with septic pulmonary emboli; stage IV lung cancer receiving chemotherapy; significant clinical deterioration while bacteremic, transitioned to hospice after 10 days hospitalization.

Age/Sex	Randomized Therapy	CrCl At Bacteremia Onset	In-Hospital Mortality	Pitt Score	Charlson Score	IL-10 (pg/mL)	Clinical Details
83/F	VAN	19	Yes	5	11	203	Bacteremia developed 30 days into hospital stay, CVL-associated septic thrombophlebitis, bacteremia persisted 5 days, ultimately cleared on VAN; died 2 days after bacteremia clearance
92/F	VAN	16	Yes	1	7	345	TAVR and ICD associated aortic valve endocarditis; 5 days bacteremia with VAN, salvaged by DAP+CPT but deteriorated rapidly while bacteremic, died hospital day 7.
70/M	VAN	44	Yes	0	6	20	Mycotic aortic aneurysm; secondary empyema; bacteremia cleared with DAP+CPT salvage after 5 days of persistence on VAN. Deemed not a surgical candidate due to rapidly declining medical condition, continued to decline, died on hospital day 29.
83/F	VAN	18	Yes	1	8	6	Prosthetic aortic valve endocarditis with multiple CNS emboli, died hospital day 7
66/M	DAP	33	Yes**	1	11	9	LVAD with newly diagnosed stage IV lung CA (not received chemotherapy yet), admitted with MRSA bacteremia from infected LVAD; daptomycin day 1-5, ceftaroline x 4 more weeks; continued to medically decline in hospital and died in hospital secondary to HCAP

Age/Sex	Randomized Therapy	CrCl At Bacteremia Onset	In-Hospital Mortality	Pitt Score	Charlson Score	IL-10 (pg/mL)	Clinical Details
56/M	Combo	57	No	8	3	102	Native aortic valve endocarditis with secondary meningitis, CNS emboli with brain abscesses, septic shoulder arthritis; Discharged after 14 days in hospital to receive ceftaroline monotherapy x 30 days.
68/F	Combo	6	No	0	8	28	ICD source; salvaged device, dialysis fistula not infected; DAP+CPT in hospital 11 days, discharged on VAN x 6 weeks at hemodialysis
79/F	Combo	40	No	3	7	5	LVAD; MRSA bacteremia involved in study enrollment was a recurrence of prior bacteremia 3 months prior; treated 7 days with DAP+CPT, discharged on DAP alone x 6 weeks followed by minocycline oral suppression; despite minocycline, recurred at 90 days, this time received DAP+CPT x 8 weeks and remained without recurrence at 6 months on oral suppressive minocycline
85/F	Combo	19	No	1	8	18	MRSA mitral valve endocarditis with lumbar spine diskitis/osteomyelitis; DAP+CPT 11 days in hospital, discharged on DAP; developed eosinophilic PNA; changed to ceftaroline to complete 3 additional weeks, then po minocycline suppression x 4 months; No recurrence, alive 6 months

Age/Sex	Randomized Therapy	CrCl At Bacteremia Onset	In-Hospital Mortality	Pitt Score	Charlson Score	IL-10 (pg/mL)	Clinical Details
74/F	Combo	8	No	0	8	71	Nursing home dialysis patient, morbid obesity; 5 days persistent bacteremia unknown source presumed endovascular; after 10 days DAP+CPT; discharged on vanco at HD x 6 weeks
71/F	Combo	87	No	2	5	12	Neck abscess, mediastinitis, septic emboli to lung, worsening mitral valve regurgitation without vegetation on 4 week echocardiogram suspecting MV endocarditidis; DAP+CPT 14 days; CPT 10 days; telavancin 8 days; tedizolid 10 days. Alive 6 months post therapy
56/F	Combo	17	No	0	10	25	Dialysis AV graft infection, end-stage cirrhosis, bacteremia 2 days
58/M	Combo	112	No	1	2	<5	Right-sided endocarditis in IVDA, Hepatitis C, bacteremia 4 days

*Died within 90 days due to *E.coli* HCAP during subsequent re-admission
**Died due to HCAP during same admission after prolonged hospital stay

VAN: Vancomycin; DAP: Daptomycin; CPT: Ceftaroline; CVL: central venous line; HCAP: health care-associated pneumonia
TAVR: trans-aortic valve replacement; ICD: implantable cardiac defibrillator; LVAD: left ventricular assist device;
CNS: central nervous system; PNA: pneumonia; HD: hemodialysis; IVDA: intravenous drug abuse; AV: arteriovenous
